# Effect of Insufficient Interaction on the Evaluation of Anesthesiologists’ Quality of Clinical Supervision by Anesthesiology Residents and Fellows

**DOI:** 10.7759/cureus.23500

**Published:** 2022-03-26

**Authors:** Rachel A Hadler, Franklin Dexter, Bradley J Hindman

**Affiliations:** 1 Anesthesia, University of Iowa, Iowa City, USA

**Keywords:** psychometrics, personnel management, teaching, hospitals, anesthesiology

## Abstract

Introduction

In this study, we tested whether raters’ (residents and fellows) decisions to evaluate (or not) critical care anesthesiologists were significantly associated with clinical interactions documented from electronic health record progress notes and whether that influenced the reliability of supervision scores. We used the de Oliveira Filho clinical supervision scale for the evaluation of faculty anesthesiologists. Email requests were sent to raters who worked one hour or longer with the anesthesiologist the preceding day in an operating room. In contrast, potential raters were requested to evaluate all critical care anesthesiologists scheduled in intensive care units during the preceding week.

Methods

Over 7.6 years, raters (N=172) received a total of 7764 requests to evaluate 21 critical care anesthesiologists. Each rater received a median/mode of three evaluation requests, one per anesthesiologist on service that week. In this retrospective cohort study, we related responses (2970 selections of “insufficient interaction” to evaluate the faculty, and 3127 completed supervision scores) to progress notes (N=25,469) electronically co-signed by the rater and anesthesiologist combination during that week.

Results

Raters with few jointly signed notes were more likely to select insufficient interaction for evaluation (P < 0.0001): 62% when no joint notes versus 1% with at least 20 joint notes during the week. Still, rater-anesthesiologist combinations with no co-authored notes accounted not only for most (78%) of the evaluation requests but also most (56%) of the completed evaluations (both P < 0.0001). Among rater and anesthesiologist combinations with each anesthesiologist receiving evaluations from multiple (at least nine) raters and each rater evaluating multiple anesthesiologists, most (72%) rater-anesthesiologist combinations were among raters who had no co-authored notes with the anesthesiologist (P < 0.0001).

Conclusions

Regular use of the supervision scale should be practiced with raters who were selected not only from their scheduled clinical site but also using electronic health record data verifying joint workload with the anesthesiologist.

## Introduction

In 2013, the de Oliveira Filho et al. clinical supervision scale in English was tested for use when evaluating faculty anesthesiologists providing operating room care [1,2]. Since then, our department has used the scale for daily evaluations of operating room anesthesiologists by resident physicians and fellows (i.e., raters), and weekly evaluations of the critical care anesthesiologists. However, unlike for operating room evaluations wherein email requests were sent daily only to raters who had worked for at least an hour with the anesthesiologist the preceding day, each potential rater was requested to evaluate all (e.g., three) critical care anesthesiologists scheduled in intensive care units during that week [3-5]. Raters chose whom to evaluate, selecting “insufficient” contact for the other anesthesiologists. In the current study, we tested whether raters’ decisions to evaluate (or not) critical care anesthesiologists were significantly associated with clinical interactions documented from electronic health record progress notes and whether that influenced the reliability of supervision scores.

## Materials and methods

The University of Iowa Institutional Review Board (IRB) declared that this project (no. 202102390), with retrospective review of de-identified data, does not meet the regulatory definition of human subjects research. The determination waived the requirement for IRB review and for written informed consent. The study did not involve human participants or live human tissue.

de Oliveira Filho et al. supervision scale

The critical care supervision score data analysed for this study were the following: scores on the 1-4 Likert scale for each of the nine items (Table 1), critical care anesthesiologist (N = 21) blinded without actual name, rater (resident or fellow, N = 172) also blinded, not actual name, and date requested for the weekly evaluation [1,2]. The average of the nine items’ scores was taken as each evaluation’s overall score [1,3,6].

**Table 1:** Items in the supervision scale used for the evaluation of critical care anesthesiologist faculty (each scored 1 = never, 2 = rarely, 3 = frequently, or 4 = always) de Oliveira Filho and colleagues developed their scale using a Delphi process among faculty anesthesiologists and resident physicians. Slight modifications were made to the scale to focus on critical care medicine. For item 5, “airway management” was used instead of “anesthesia induction.” For item 6, “peri-anesthesia management” was shortened to “management” and “anesthetic procedure” reduced to “procedure.” For item 7, each of the examples in the parentheses are different (e.g., removed “anesthesia machine checkout”). All items had to be answered for submission.

Sequence	Item
1	The faculty provided me timely, informal, nonthreatening comments on my performance and showed me ways to improve
2	The faculty was promptly available to help me solve problems with patients and procedures
3	The faculty used real clinical scenarios to stimulate my clinical reasoning, critical thinking, and theoretical learning
4	The faculty demonstrated theoretical knowledge, proficiency at procedures, ethical behavior, and interest/ compassion/respect for patients
5	The faculty was present during the critical moments of procedures (e.g., airway management, critical events, complications)
6	The faculty discussed with me the management of patients prior to starting a procedure or new therapy and accepted my suggestions, when appropriate
7	The faculty taught and demanded the implementation of safety measures (e.g., time outs, infection control practices, consideration of deep vein thrombosis and stress ulcer prophylaxis and patient mobilization)
8	The faculty treated me respectfully and strove to create and maintain a pleasant environment during my clinical activities
9	The faculty gave me opportunities to perform procedures and encouraged my professional autonomy

Data on requests to evaluate quality of supervision

The dates of these evaluations were for clinical time together from July 1, 2013 to February 7, 2021. Each of the evaluation requests (N = 7764) was sent on a Monday to raters who had a critical care assignment for the preceding week. Each of the four units was staffed by one critical care faculty member (anesthesiologist, emergency medicine physician, or surgeon) during standard working hours, seven days per week. Daytime faculty coauthored daily progress notes with residents in their assigned unit. Two faculty were on-call from home each night and covered their assigned unit and one other unit. Notes for patients admitted after hours were signed by the on-call faculty member. Emergency medicine and surgical critical care faculty were not evaluated by the Department of Anesthesia.

The raters (i.e., anesthesiology resident physicians and critical care fellows) assigned weekly to any of the four critical care units were sent evaluation requests for the preceding week [7]. While the anesthesiology residents would also use the supervision scale when evaluating operating room anesthesiologists and pain medicine physicians, the critical care fellows would not do so contemporaneously, but often did so the previous year if they had graduated from the program's anesthesiology residency. The email instructions read, “Dear Dr. (rater), you interacted with Dr. (ratee) for an hour or more during the week of (date) to (date). Click here to go to the Evaluation System.” Following a week with a critical care assignment, each rater received an email for each ratee (i.e., faculty member) who had been scheduled that week even though it is possible the rater might have not interacted with some of the potential ratees during the week. When the rater clicked and logged in to the secure site, the first option was to select “No evaluation because Dr. (ratee) and I were not assigned to care for patients together ≥60 minutes” [2,7]. Otherwise, all nine items in the supervision scale had to be completed for submission (Table 1). Evaluation requests not completed within two weeks were removed from raters’ lists and counted as non-response. This process matched that for the operating rooms (see Introduction), except for the small modifications of the survey items and the frequency of evaluation requests (daily vs. weekly, respectively) (Table 1). The final date of clinical time together, February 7, 2021, was a Sunday. Therefore, the final date that evaluation requests were sent was Monday, February 8, 2021, and the final date for data collection was Sunday, February 21, 2021. Table 2 summarizes the internal consistency, concurrent validity, and generalizability analysis of the scale [8-14].

**Table 2:** Internal consistency, concurrent validity, and generalizability analysis of the critical care supervision scale ^a^To interpret Cronbach’s alpha, for each respondent, four or five of the nine items in the scale were selected (Table 1), and the mean score was calculated. The mean score of the other four or five items was also calculated. Among all respondents, the correlation coefficient between the pairwise split-half mean scores was calculated. The process was repeated using all possible split-halves of the four or five items. The mean of the correlation coefficients was Cronbach’s alpha value, measuring the internal consistency of items [8]. ^b^The 95% confidence interval for the Spearman rank correlation coefficient was calculated asymptotically. The P‑value was calculated by Monte-Carlo simulation to six-digit accuracy (StatXact 12.0; Cytel, Inc., Cambridge, MA). ^c^To estimate the generalizability coefficient with different numbers of independent raters, initial conditions used for integer programming were the 3127 scored evaluations among the subset (N = 157) of raters who each had at least six anesthesiologists and the subset (N = 20) of anesthesiologists each with at least six raters, for a total of 1207 observed combinations of rater and anesthesiologist each with at least one evaluation. For each of the combinations, the mean of the average scores was used in an integer program, solved to maximize the number of combinations in a balanced design. Excel 365 Solver (Microsoft, Redmond, WA) was used with the Evolutionary solving method, automatic scaling, a mutation rate of 0.15, and a population size of 150. With multiple initial conditions and parameter values, the same solution was obtained with N = 21 raters, and N = 9 anesthesiologists. ^d^The Stata command used was gstudy (Stata 16.1; StataCorp LLC, College Station, TX).

Test	Finding	Result, calculation, and interpretation, in sequence
Internal consistency	1	Cronbach’s alpha of the 9 items equaled 0.961 (95% confidence interval 0.959-0.963) [9,10].^a^
Internal consistency	2	The sample size, N = 3127 completed (scored) critical care evaluations.
Internal consistency	3	This “excellent” result was comparable to that for the operating room supervision scale when completed by residents and fellows, Cronbach’s alpha 0.95 and 0.98 [11-13].
Concurrent validity	1	Spearman’s rank correlation between anesthesiologists’ paired average critical care evaluation supervision scores and average operating room evaluation supervision scores was 0.732 (95% confidence interval 0.406-0.999, P = 0.0027).
Concurrent validity	2	The sample size was N = 15, critical care anesthesiologists who also provided clinical anesthesia care in operating rooms [2]. There were nine items requested for evaluation, using the same date period working together [1,2]. For both supervision scales, the mean score was calculated among all evaluations of the anesthesiologist and used as a summary measure. The Spearman correlation was calculated between the pairwise combinations of means.^b^
Concurrent validity	3	The correlation was greater than observed previously [2,13,14].
Generalizability coefficient	1	With the largest possible fully crossed design (21 raters and 9 anesthesiologists), the estimated generalizability coefficient was 0.65 (i.e., ≈101 raters to obtain G-coefficient 0.90).^c^
Generalizability coefficient	2	The analysis of variance method was used to estimate this relative generalizability coefficient for the one facet completely crossed design.^d^
Generalizability coefficient	3	This low generalizability coefficient finding prompted the current study.

Data on progress notes

The other data used were counts of the 25,469 Epic progress notes (Epic, Verona, WI) electronically signed both by a rater and ratee during an evaluation week. Table 3 summarizes the distribution of the progress notes among the 7764 evaluation requests. Most raters had no notes in common with more than one anesthesiologist working during the same week. Consequently, the notes matched how the rater and anesthesiologist assignments were made, just not recorded reliably in departmental staff scheduling tables over the study period.

**Table 3:** Distribution of jointly signed progress notes among rater and ratee (critical care anesthesiologist) combinations

Sorting N = 7764 evaluation requests by week, then by rater, and then in a descending sequence among ratees in the counts of jointly signed progress notes	Number of combinations of the rater and ratee	Mean (standard deviation) of progress notes for the week	90th percentile of progress notes for the week
Rater and ratee combinations with the largest number of jointly electronically signed progress notes that week	2697	9.17 (9.28)	22
Rater and ratee combinations with the second largest number of jointly electronically signed progress notes that week	2583	0.27 (1.64)	0
Rater and ratee combinations with the third largest number of jointly electronically signed progress notes that week	1909	0.02 (0.23)	0
Rater and ratee combinations with the fourth largest number of jointly electronically signed progress notes	493	0.01	0
Rater and ratee combinations with the fifth largest number of jointly electronically signed progress notes	82	0	0

The observed progress notes signed both by the rater and anesthesiologist were used to create five categories, shown as the headers of Table 4. The five sequential categories were selected using integer programming based on minimizing the root mean square differences in sample sizes of evaluation requests among categories.

**Table 4:** Relationship between endpoints and ordered categories of numbers of patient progress notes signed electronically during the week both by the rater (i.e., resident or fellow) and ratee (i.e., critical care anesthesiologist) ^a^To interpret the 7764 requests, among the 397 studied weeks, there were mean 6.8 (SD 2.8) anesthesiology resident physicians and critical care fellows assigned weekly to the surgical and neurological intensive care unit or cardiac intensive care unit. Each rater received email requests to evaluate a mean of 3.0 (SD 0.8) faculty (potential ratees) from the week. The product of 397 weeks, 6.8 potential raters per week, and 3.0 potential ratees per week does not equals 7764 because the mean is being taken for each rater. ^b^The percentages of evaluations returned, with “insufficient” or with the nine items scored, are compared with 50%, for testing “most”. These two percentages are labeled b. The 95% confidence interval for 6019/7764 is 77% to 78%. The 95% confidence interval for 1756/3127 is 54% to 58%.

Variable segmented by categories of notes completed jointly with the faculty ratee	0	1 to 8	9 to 14	15 to 19	20 to 42	Cochran-Armitage trend test of the row
Requests for evaluation (% of the 7764 requests)^a^	6019 (78%)^b^	392 (5%)	449 (6%)	473 (6%)	431 (6%)	
Responses, either “insufficient” or the 9 items completed (% of column Requests for evaluation, from the preceding row)	4677 (78%)	306 (78%)	379 (84%)	388 (82%)	347 (81%)	0.0012
Insufficient given as response (% of column Responses, from the preceding row)	2921 (62%)	21 (7%)	12 (3%)	11 (3%)	5 (1%)	<0.0001
Evaluations completed (% of the 3127 evaluations)	1756 (56%)^b^	285 (9%)	367 (12%)	377 (12%)	342 (11%)	
Evaluations with all nine items given scores of 4 (% of column Evaluations completed, from the preceding row)	1292 (74%)	186 (65%)	259 (71%)	277 (73%)	261 (76%)	0.61

Analysis of the validity of the use of progress note counts

Discriminant validity was tested by checking for the absence of an association between rater-anesthesiologist interaction and evaluation scores, because few progress notes in common do not necessarily mean the absence of supervision but that the rater and anesthesiologist were principally responsible for different patients. Consistently, at least half of supervision scores equaled 4.00 (i.e., all nine items were given scores of 4) [3,4,6,13]. Therefore, the Cochran-Armitage trend test was used to evaluate the ordered association between the five categories of the numbers of progress notes signed by the rater and anesthesiologist and the percentages of rater scores equaling 4.00 (Table 4). The two-sided P-value was calculated using Monte-Carlo simulation (StatXact 12.0; Cytel, Inc., Cambridge, MA).

Concurrent validity was evaluated by checking for a weak but positive association between the counts of jointly signed progress notes and response rate. Concordance was expected because not responding could be considered complementary to logging in and entering insufficient interaction. An inverse association was expected between weekly counts of joint progress notes and the percentage of evaluations returned with reported insufficient interaction. The Cochran-Armitage trend test was used, calculated using StatXact.

Analysis of raters’ decisions on whether to evaluate and their associations with progress notes

The primary question of managerial importance was whether there were many evaluations completed by raters with no detectable interaction with the anesthesiologist. The exact binomial test was whether that was so for most (greater than half) evaluations. The Clopper-Pearson method was used to calculate the 95% two-sided confidence interval for the percentage. Both were calculated using StatXact. The same approach was used to test whether most of the analyzable combinations of raters and anesthesiologists were among pairs with no progress notes jointly signed during the week.

There was no a priori sample size analysis, as all data available were used, and the study was started corresponding with the retirement of the faculty responsible for the evaluation program. Therefore, our primary question was limited to statements with corresponding lower confidence limits reliably greater than would be managerially important.

## Results

The evaluation response rate was 79%, with 49% of the responses reported as insufficient interaction, and the other 51% being completed (scored) evaluations (Table 5).

**Table 5:** Relationship between endpoints and cumulative counts of patient progress notes signed electronically during the week both by the rater (i.e., resident or fellow) and ratee (i.e., critical care anesthesiologist) ^a^The categories were created cumulatively from those in Table 4, selected using integer programming based on minimizing the root mean square differences in sample sizes of evaluation requests among categories. ^b^The evaluation response rate of 79% = 6097/7764 (in the preceding row of the same column). Among the 172 raters, there were 14 with at least 100 requests. They had a mean (SD) response rate of 79% (20%). Therefore, there was considerable heterogeneity of response rates among raters. ^c^Raters averaged 9.17 notes per week with one faculty (ratee) and only 0.27 notes per week with the faculty with the second-largest number of notes jointly with the rater (Table 3). Also, there was a mean of 2.9 invitations per week, with mode 3.0 invitations and median 3.0 invitations. Therefore, the expected percentage of “insufficient” would have been approximately 2/3 (i.e., on evaluating the 1 of 3 faculty with whom the rater worked regularly), not 49% in the cell labeled c. In addition, there was heterogeneity in this reported 49% among raters. Specifically, among the 172 raters, there were 11 with at least 100 responses. They had mean (standard deviation) percentage insufficient of 39% (24%). ^d^We found that “most (72%) of the usable combinations of raters and anesthesiologists were among raters who had no notes signed jointly with the ratee.” Most (i.e., 50%) is being compared with the percentage of rater and ratee combinations, limited exclusively to weeks with no progress notes jointly signed. The counts used are labeled d. The reported 72% = (610 – 172)/610. The 95% confidence interval is 68% to 75%.

Variable versus cumulative counts of notes completed jointly with the faculty ratee	0 to 42^a^	1 to 42^a^	9 to 42^a^	15 to 42^a^
Requests for evaluation (% of the 7764 requests)	7764 (100%)	1745 (22%)	1353 (17%)	904 (12%)
Responses, either “insufficient” or the 9 items completed (% of column Requests for evaluation, from the preceding row)	6097 (79%)^b^	1420 (81%)	1114 (82%)	735 (81%)
Insufficient given as response (% of column Responses, from the preceding row)	2970 (49%)^c^	49 (3%)	28 (3%)	16 (2%)
Evaluations completed (% of the 3127 evaluations)	3127 (100%)	1371 (44%)	1086 (35%)	719 (23%)
Evaluations with score 4.00 was mean of the 9 items (% of column Evaluations completed, from the preceding row)	2275 (73%)	983 (72%)	797 (73%)	538 (75%)
Evaluations completed excluding raters with all scores at 4.00 (% of the 3127 evaluations)	2652 (85%)	1066 (34%)	819 (26%)	451 (14%)
Rater and ratee combinations with ≥1 evaluation, excluding raters with all scores at 4.00 (% of the 1207 combinations)	1004 (83%)	660 (55%)	556 (46%)	335 (28%)
Rater and ratee combinations, excluding raters with all scores at 4.00, raters with <9 ratees, and ratees <9 raters (% of the 1207 combinations)	610 (51%)^d^	172 (14%)^d^	86 (7%)	0 (0%)

Validity of the use of counts of progress notes

Respondents with fewer anesthesiologist interactions based on joint progress notes had a slightly but significantly lower response rate (P = 0.0012): 78% among raters with no joint notes during the week versus 81% among raters with at least 20 joint notes (Table 4). Respondents with fewer jointly signed notes were more likely to select insufficient interaction for evaluation (P < 0.0001): 62% when no joint notes versus 1% with at least 20 joint notes during the week (Table 4). These two results show concurrent validity of analysing the counts of jointly signed notes.

There was no association between counts of progress notes electronically signed by raters and anesthesiologists during the week and the percentage of the evaluations with the largest score (P = 0.61; Table 4). This finding shows the discriminant validity of using the counts of joint notes.

Primary analyses: raters’ decisions on whether to evaluate and their associations with progress notes

Rater and anesthesiologist combinations with not a single (zero) progress note signed jointly accounted not only for the most (78%) evaluation requests but also the most (56%) completed evaluations (both P < 0.0001; Table 4). Functionally, an evaluation system’s sample size is the number of rater and anesthesiologist combinations among anesthesiologists receiving evaluations from multiple (e.g., at least nine) raters and among raters providing different scores among multiple anesthesiologists. Most (72%) of the usable combinations of raters and anesthesiologists were among raters who had no notes signed jointly with the ratee (P < 0.0001; Table 5).

## Discussion

The quality of anesthesiologists’ supervision matters because it is an independent contributor to faculty members’ clinical value, separate from the amount of clinical care provided (e.g., as measured using clinical days worked) [15]. In addition, the quality of supervision is an integral component of the professionalism of anesthesiologists [16]. Our current study reports on raters’ behaviors (i.e., resident physicians and fellows) when requested to evaluate faculty with whom they may have had little interaction. Although raters often recorded there had been insufficient interaction for evaluation (as expected), because most requests were made when the documented anesthesiologist-rater interaction was low, more than half the evaluations were completed under these conditions. Specifically, and importantly, from the 95% confidence intervals, at least two-thirds of the rater and anesthesiologist combinations with completed evaluations probably were completed by the raters despite little clinical interaction (Table 4). The results show that the integrity of the process of evaluating anesthesiologists using the de Oliveira Filho et al. supervision scale should not be assumed to be acceptable based solely on the numbers of raters per anesthesiologist, because that is a necessary but insufficient endpoint.

The implications are large for an appropriate use of the de Oliveira Filho et al. supervision scale when used for daily or weekly evaluations [1]. Previously, anesthesia residents’ evaluations of anesthesiologists were studied in the operating room environment [4,17,18]. Requests for residents’ evaluations of anesthesiologists’ quality of supervision were sent only if they worked together that day for at least one hour [3-5]. Our study suggests that this rater selection method, which the investigators had considered as a means to reduce the inconvenience to potential raters, probably was an integral process requirement for the scale’s success in the operating room, necessary for psychometric reliability [3-6,17,18]. Although one hour per day for the operating rooms may appear brief, a comparable threshold for critical care unit evaluations would (because of trainee work rules) be six hours for the week, where six hours = one hour per day × six days over the week. As recommended in a systematic review, it is important to include an option to select “insufficient interaction,” or the equivalent [7]. However, our results show that this “insufficient interaction” option should be for a small percentage of residual interactions​​​​​, not replacing the use of electronic health record data for rater selection [3,12,16,17,19].

Limitations

We are unaware of the earlier work on the choice to evaluate faculty based on the invitation process. Our study was performed at one large teaching hospital, albeit with a typical set of surgical and cardiac intensive care units. The inability therefore to compare our results with other papers is a limitation but highlights the novelty and importance of our work. Although we have data only for use with the de Oliveira Filho et al. supervision scale, we would expect that the findings would apply more broadly to daily or weekly clinical evaluations of faculty.

Although we considered the evaluation of anesthesiologists, results may apply to evaluation of other anesthesia practitioners, in countries where relevant. For example, requests to anesthesiologists to evaluate nurse anesthetists’ work habits were sent only if the anesthesiologist and nurse anesthetist both started at least one case together and worked together that day for at least one hour [4,17,18]. Our results suggest this process be considered an important component of such a program.

Our results do not supply evidence one way or the other as to whether the supervision scale should be used for evaluating individual performance of critical care anesthesiologists at supervising resident physicians and critical care fellows. We hypothesize that evaluations based on low levels of the rater-ratee interaction caused the low generalizability coefficient (i.e., insufficient reliability of the supervision scale for the evaluation of individual critical care anesthesiologists) (Table 2). However, we cannot test this hypothesis with the available data because deleting all rater and anesthesiologist combinations with no co-authored progress notes results in too few paired raters and ratees (Table 5) for generalizability analysis (Table 2). When interaction was limited but evaluation submitted, raters might have relied on the overall quality of supervision obtained during the week by all personnel, including other trainees, experienced nurses, and consulting physicians (e.g., palliative care or infectious disease specialists).

For evaluations of chronic pain medicine faculty using the de Oliveira Filho et al. supervision scale, raters’ use of “insufficient contact” (11%) was much less than that for critical care (49%) [13]. Differences in the raters’ use of “insufficient contact” were probably because the daily pain clinic arrangement was one faculty with one resident or fellow for the day, more like in an operating room environment.

Although our available electronic health record data used to measure the rater and anesthesiologist relationship in critical care was crude, our results showed both concurrent and discriminant validity. Nevertheless, brief trainee-faculty interactions are common in clinical care and do not imply a lack of substantive interaction. Relying on the rater’s judgement to determine the degree of the interaction is important and necessary because availability and physical presence are integral components of supervision [12,16]. However, this cannot change our results and their implications, specifically previous reports, of the supervision scale being reliable and valid in the operating room setting when applied daily should be considered limited to the precise process that was used, with invitations to evaluate anesthesiologists sent only with documentation of the joint workload in the electronic health record [3,5,6,12,15,16].

## Conclusions

Evaluation requests were sent for all potential rater-anesthesiologist interactions. Our results show that this resulted in many evaluations being made by raters unlikely to have had a close interaction with the anesthesiologist over the week. Low-contact interactions constituted most of the requested and completed evaluations. The implication of our results is that wherever the quality of an anesthesiologist’s supervision care is evaluated on a daily or weekly basis using the de Oliveira Filho et al. instrument, staff scheduling data alone should be considered insufficient for selecting potential raters. Instead, the degree of rater-anesthesiologist interactions needs to be measured by using electronic health record data and to be used to select raters to whom to send evaluation requests.
